# Human immunodeficiency virus-1 genome from patient with fever, Nepal

**DOI:** 10.1128/mra.00768-24

**Published:** 2024-10-21

**Authors:** Eans Tara Tuladhar, Bimal Sharma Chalise, Binod Khadka, Mamta Tamang, Jenish Neupane, Shankar Poudel, Lindsay Droit, Kathie A. Mihindukulasuriya, Annie Elong Ngono, Yuba Nidhi Basaula, Sujan Shresta, David Wang, Krishna Das Manandhar

**Affiliations:** 1 Tribhuvan University Central Department of Biotechnology, Kathmandu, Nepal; 2 Tribhuvan University Maharajgunj Medical Campus, Kathmandu, Nepal; 3 Sukraraj Tropical and Infectious Disease Hospital, Kathmandu, Nepal; 4 Washington University School of Medicine in St. Louis, St. Louis, Missouri, USA; 5 La Jolla Institute for Immunology, La Jolla, California, USA; DOE Joint Genome Institute, Berkeley, California, USA

**Keywords:** HIV, fever of unknown origin, WGS

## Abstract

A patient with fever presented to the referral infectious disease hospital in Kathmandu, Nepal. Metagenomic sequencing of the patient’s serum recovered a near-complete genome of human immunodeficiency virus-1 (HIV-1), distinct from previous HIV-1 genomes from Nepal in GenBank. It shared 92.48% nucleotide identity with an HIV-1 subtype C isolate from India.

## ANNOUNCEMENT

Human immunodeficiency virus-1 (HIV-1) is a member of the genus *Lentivirus* in the family *Retroviridae*. GenBank contains only a limited number of near-complete HIV-1 genome sequences from Nepal. The most recent was reported in 2020 from samples collected in 2015.

**Fig 1:**
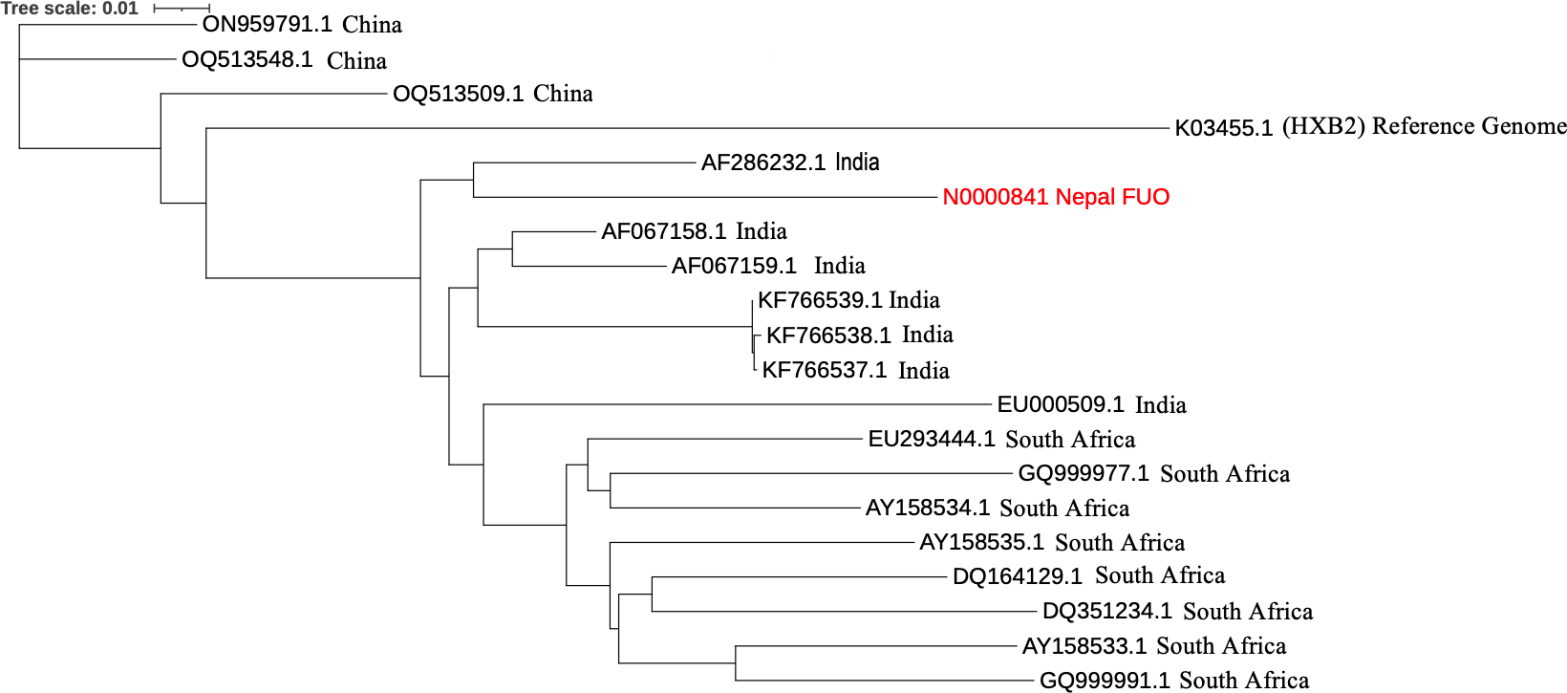
Maximum likelihood phylogenetic tree of the Nepalese HIV-1 isolate reported here (N0000841) with 18 complete genome sequences with the highest percentage identity and a reference genome. N0000841 lies in the HIV-1 clade reported from India.

A 51-year-old male presented with fever of unknown origin (FUO) at Sukraraj Tropical and Infectious Disease Hospital (STIDH), Kathmandu, Nepal, in June 2023. Total nucleic acids were extracted from the patient’s serum using an Invitrogen Pure Link Viral RNA/DNA Mini Kit (Thermo Fisher Scientific) following the instruction manual and eluted in 50 µL RNase-free water. cDNA was obtained by reverse transcription using M-MLV Reverse Transcriptase (Promega) with a 31-base random primer (TACCGTAGAGCTGCTANNNNNNNNNNNNNNN). This was followed by second-strand synthesis with Sequenase Enzyme (Applied Biosystems, Thermo Fisher Scientific) and PCR amplification using a primer consisting of the first 16 bases of the reverse transcription primer (TACCGTAGAGCTGCTA) (1, 2). A library was constructed using the Illumina DNA Prep kit and sequenced (2 × 150 bp paired-end) on the Illumina NextSeq 500. A total number of 5,019,323 reads were obtained. Next generation sequencing (NGS) data were analyzed for the presence of viral sequences using the cloud-based platform CZ ID (3). Many HIV reads were detected; therefore, MEGAHIT v.1.2.9 (4) was used to assemble the raw reads, generating multiple contigs, of which one, 8,792 nucleotides long contig (N0000841 Nepal FUO) with an average read depth of 7×, mapped to HIV-1. The average length of the individual HIV sequence reads was 150 nucleotides. The GC content of the contig was 41.47%. The contig shared 92.48% identity with the HIV-1 subtype C isolate reported from India (accession # AF067158.1) via NCBI blastn (Date of access: 5 June 2024). VAPiD (5) v.1.6.7 was used for annotation with AF067158.1 as the reference genome. A maximum likelihood phylogenetic tree was generated with the 18 whole genome sequences of HIV-1 with highest identity % blastn scores downloaded from NCBI (accessed on 5 June 2024) with 1,000 bootstrap replications using W-IQ-TREE (6) and visualized using iTOL (7) (Fig. 1). The multiple sequence alignment was performed using Clustal Omega (8). Default parameters were used except where otherwise noted. There were 465 publicly accessible GenBank HIV-1 genome sequences (including 14 partial genomes of >8,000 bp) from Nepal (accessed on 5 June 2024) using search phrase “HIV Nepal.” Among Nepalese HIV sequences, online NCBI blastn algorithm pipeline with default parameters demonstrated the highest identity (91.91%) to HIV-1 isolate NP20 collected in 2015 (accession # MK493077.1). This study informs our understanding of the current status of the HIV-1 genome diversity in the Nepalese population.

## Data Availability

The genome assembly (N0000841 Nepal FUO) has been deposited in GenBank, accession # PP879189. The version described in this paper is the first version PP879189.1. The raw reads have been submitted to SRA: SRS22420834 under BioProject PRJNA1148996 with BioSample: SAMN43226821.
